# Supplementary material for: Fluorescence‐based CRISPR interference system for controlled genetic repression and live single‐cell imaging in mycobacteria
Source: FEBS Lett. 2024 Dec 1;599(4):488–501. doi: 10.1002/1873-3468.15071 (PMC11848015; doi:10.1002/1873-3468.15071)
Supplement: Supplementary file 1 — Fig. S1. Generation and functional validation of pJL29 and pJL30 fluorescent vectors. Fig. S2. Analysis of pJL32‐mediated fluorescence in comparison to its parental pIRL117 vector on agar and in liquid medium. Fig. S3. Comparison of pIRL117‐ and pJL32‐mediated targeting of essential genes in M. smegmatis. Fig. S4. Illustration of the one‐step co‐transformation experiment performed in M. tuberculosis. Table S1. List of primers used in this study. Table S2. List of the plasmids used in this study. [file FEB2-599-488-s001.pdf]

Supporting information

Additional supporting information may be found online in the Supporting Information section at the end of the article.

Fluorescence-based CRISPR interference system for controlled genetic repression and live single-cell imaging in mycobacteria

Janïs Laudouze[1#], Vanessa Point[1#], Wafaa Achache[1,2], Céline Crauste[3], Stéphane Canaan[1] & Pierre Santucci[1*]

[1] Aix Marseille Univ, CNRS, LISM, IMM FR3479, IM2B, Marseille, France

[2] IHU Méditerranée Infection, Aix-Marseille Univ., France.

[3] IBMM, Univ Montpellier, CNRS, ENSCM, 34000 Montpellier, France.

[#] Contributed equally as co-first authors

* Correspondence address to Pierre Santucci, psantucci@imm.cnrs.fr

ORCID https://orcid.org/0000-0002-6291-3425

Table S1. List of the primers used in this study.

Table S1. Primers used in this study			
**Number**	**Name**	**Restriction site** (if applicable)	**Sequence 5'-3'**
#P1	pUV15_mWasabi_Fwd	SphI	TATAGCATGCTTAATTAAGGAGGCACCGGTATGGTGAGCAAGGGCGAGGAG
#P2	pUV15_mWasabi_Rev	HindIII	TATAAAGCTTTTACTTGTACAGCTCGTCCATGC
#P3	pUV15_dtomato_Fwd	SphI	TATAGCATGCTTAATTAAGGAGGCACCGGTATGGTGAGCAAGGGCGAGGAG
#P4	pUV15_dtomato_Rev	HindIII	TATAAAGCTTCTACTTGTACAGCTCGTCCATGC
#P5	pIRL_psmyc_Fluo_Fwd	-	TATAGAATTCGCGTTTAATACTGTTTAAACTCTAG
#P6	CRISPRi-pIRL-Sequencing	-	TTCCTGTGAAGAGCCATTGATAATG
#P7	pIRL_RpoB_MSMEG_1367_Top	BsmBI-Cut	GGGAGACCTCGTCGAAGCGCGGATC
#P8	pIRL_RpoB_MSMEG_1367_Bottom	BsmBI-Cut	AAACGATCCGCGCTTCGACGAGGTC
#P9	pIRL_MmpL3_MSMEG_0250_Top	BsmBI-Cut	GGGAATCACGAGCGTGAGCGGTTCGGT
#P10	pIRL_MmpL3_MSMEG_0250_Bottom	BsmBI-Cut	AAACACCGAACCGCTCACGCTCGTGAT
#P11	pIRL_MmpL4b_MSMEG_0382_Top	BsmBI-Cut	GGGAGTCCCGCACTCTGCAGGCTCA
#P12	pIRL_MmpL4b_MSMEG_0382_Bottom	BsmBI-Cut	AAACTGAGCCTGCAGAGTGCGGGAC

Table S2. List of the plasmids used in this study.

Table S2. Plasmids used in this study			
**Name**	**Alternative Name**	**Short Description**	**Reference and/or Source**
pTEC15	-	Hygromycin resistant episomal vector for the constitutive expression of mWasabi under the control of the *MSP12* promotor (pMSP12::mWasabi)	Takaki *et al.* 2013 - Addgene Plasmid #30174
pTEC27	-	Hygromycin resistant episomal vector for the constitutive expression of dTomato under the control of the *MSP12* promotor (pMSP12::dTomato)	Takaki *et al.* 2013 - Addgene Plasmid #30182
pUV15-pHGFP	-	Hygromycin resistant episomal vector for the constitutive expression of pHGFP under the control of the *psmyc* promotor (psmyc::pHGFP)	Vandal *et al.* 2008 - Addgene Plasmid #70045
pJL29	pUV15_mWasabi	Hygromycin resistant episomal vector for the constitutive expression of mWasabi under the control of the *psmyc* promotor (psmyc::mWasabi)	This study - Addgene Plasmid #227426
pJL30	pUV15_dTomato	Hygromycin resistant episomal vector for the constitutive expression of dTomato under the control of the *psmyc* promotor (psmyc::dTomato)	This study - Addgene Plasmid #227427
pIRL2	-	Kanamycin resistant L5-integrative vector for CRISPR interference in *M.tuberculosis*	Bosch *et al.* 2020 - Addgene Plasmid #163631
pIRL117	-	Kanamycin resistant L5-integrative vector for CRISPR interference in *M.smegmatis*	Bosch *et al.* 2020 - Addgene Plasmid #163635
pJL31	pIRL117_*psmyc::mWasabi*	Kanamycin resistant L5-integrative vector for CRISPR interference in *M.smegmatis* with constitutive expression of mWasabi under the control of the psmyc promotor (psmyc::mWasabi)	This study - Addgene Plasmid #227428
pJL32	pIRL117_*psmyc::dTomato*	Kanamycin resistant L5-integrative vector for CRISPR interference in *M.smegmatis* with the constitutive expression of dTomato under the control of the psmyc promotor (psmyc::dTomato)	This study - Addgene Plasmid #227429
pJL33	pIRL2_*psmyc::mWasabi*	Kanamycin resistant L5-integrative vector for CRISPR interference in *M.tuberculosis* with the constitutive expression of mWasabi under the control of the psmyc promotor (psmyc::mWasabi)	This study - Addgene Plasmid #227430

pJL34	pIRL2_*psmyc::dTomato*	Kanamycin resistant L5-integrative vector for CRISPR interference in *M.tuberculosis* with the constitutive expression of dTomato under the control of the psmyc promotor (psmyc::dTomato)	This study - Addgene Plasmid #227431
pJL1	pIRL117_*rpoB*	Kanamycin resistant L5-integrative vector for CRISPR interference of *rpoB* in *M.smegmatis*	This study
pJL2	pIRL117_*mmpL3*	Kanamycin resistant L5-integrative vector for CRISPR interference of *mmpL3* in *M.smegmatis*	This study
pJL3	pIRL117_*mmpL4b*	Kanamycin resistant L5-integrative vector for CRISPR interference of *mmpL4b* in *M.smegmatis*	This study
pJL35	pJL32_*rpoB*	Kanamycin resistant L5-integrative vector for CRISPR interference of *rpoB* in *M.smegmatis* with the constitutive expression of dTomato under the control of the psmyc promotor (psmyc::dTomato)	This study
pJL36	pJL32_*mmpL3*	Kanamycin resistant L5-integrative vector for CRISPR interference of *mmpL3* in *M.smegmatis* with the constitutive expression of dTomato under the control of the psmyc promotor (psmyc::dTomato)	This study
pJL37	pJL32_*mmpL4b*	Kanamycin resistant L5-integrative vector for CRISPR interference of *mmpL4b* in *M.smegmatis* with the constitutive expression of dTomato under the control of the psmyc promotor (psmyc::dTomato)	This study

Figure S1. Generation and functional validation of pJL29 and pJL30 fluorescent vectors. (A) Schematic representation of the original pTEC15, pTEC27 and pUV15-pHGFP vectors and the newly generated pJL29 and pJL30 vectors harbouring the *mWasabi* or *dTomato* coding sequence under the control of the strong constitutive *psmyc* promotor. **(B-C)** Detection and comparative analysis of *M. smegmatis* recombinant strains harbouring the original pTEC15, pTEC27 construct or the pJL29 or pJL30 vectors when plated onto 7H10 agar plates. The pMV306-Hyg was used as fluorescent-negative control. Bright light, green fluorescence, red fluorescence, merge micrographs are displayed. **(D-E)** Analysis of pJL-mediated fluorescence in comparison to its parental pTEC vectors on agar and in liquid medium. Fluorescence display of *M. smegmatis* recombinant strains when spotted as macro-colonies on 7H10 agar plates (bottom panel) or when cultured in 7H9 broth media (top panel).

Figure S2. Analysis of pJL32-mediated fluorescence in comparison to its parental pIRL117 vector on agar and in liquid medium. (A) Fluorescence display of *M. smegmatis* pIRL117 and pJL32 recombinant strains when spotted as macro-colonies on 7H10 agar plates. **(B)** Fluorescence display of *M. smegmatis* pIRL117 and pJL32 recombinant strains when cultured in 7H9 broth media.

Figure S3. Comparison of pIRL117- and pJL32-mediated targeting of essential genes in *M. smegmatis*. (A) Functional validation of pJL32 CRISPRi system by targeting *rpoB*, *mmpL3*, and *mmpL4b*. Serial dilution of *M. smegmatis* recombinants strains was spotted onto 7H10 agar media in the absence ATc (Left panel) or in the presence ATc (Right panel) of 100 ng/mL of anhydrotetracycline. Bright light (top) and their corresponding red fluorescent profiles (bottom) are displayed. **(B)** Functional validation and comparison of pIRL117 CRISPRi system by targeting *rpoB*, *mmpL3*, and *mmpL4b*. Serial dilution of *M. smegmatis* recombinant strains was spotted onto 7H10 agar media in the absence ATc (Left panel) or in the presence ATc (Right panel) of 100 ng/mL of anhydrotetracycline. Bright light (top) and their corresponding red fluorescent profiles (bottom) are displayed. Non-fluorescent recombinant strains display a very low fluorescence signal which is as comparable as the background intensity from the medium.

Figure S4. Illustration of the one-step co-transformation experiment performed in *M. tuberculosis*. (A) Fluorescence display of *M. tuberculosis* pJL29, pJL34 or pJL29/34 recombinant strains when selected post-electroporation onto 7H10 agar plates. Bright light, red fluorescence, green fluorescence and merge micrographs are displayed from left to right.

Figure S1

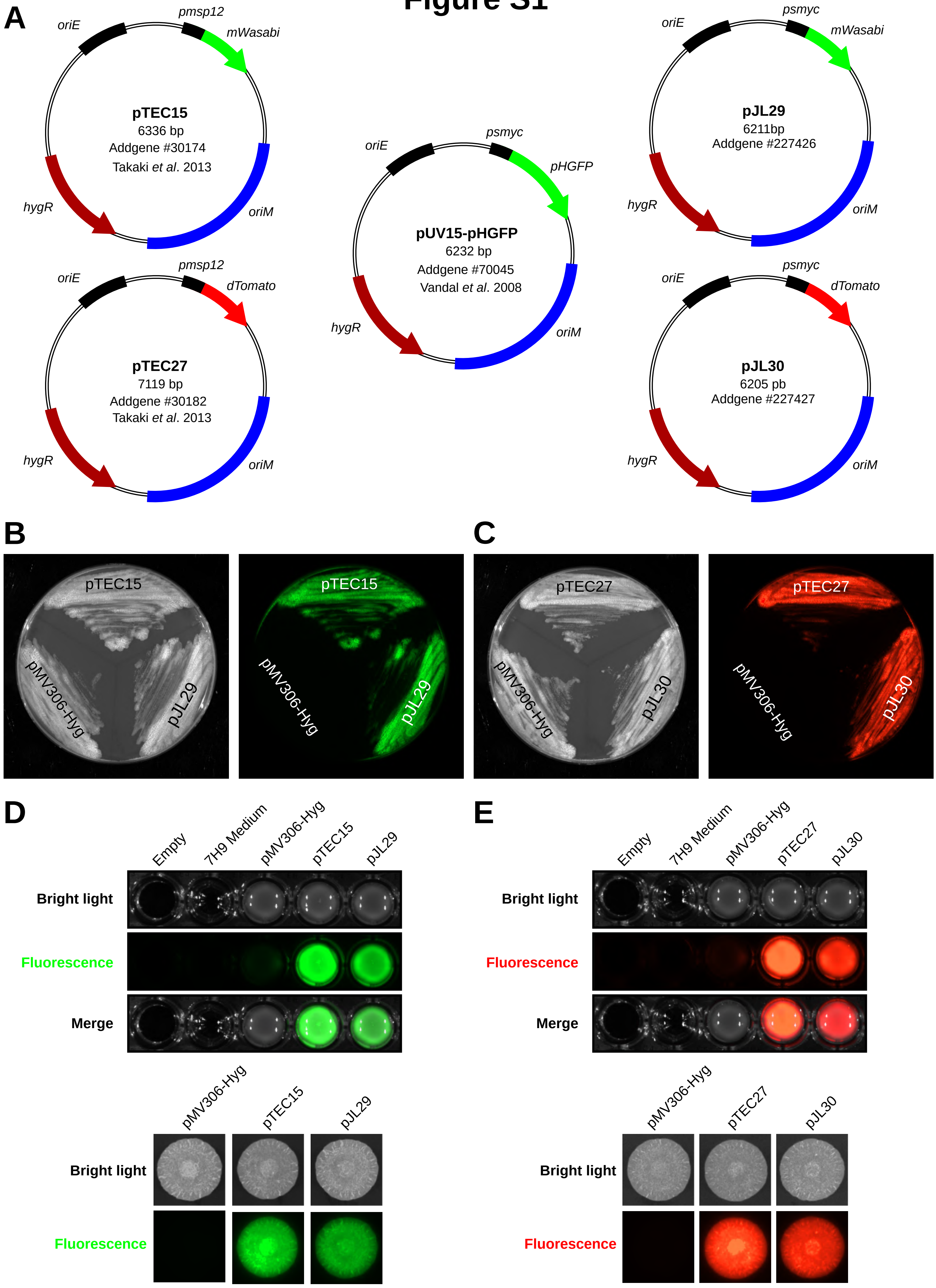

A
oriE
pmsp12
mWasabi
pTEC15
6336 bp
Addgene #30174
Takaki et al. 2013
hygR
oriM
pTEC27
7119 bp
Addgene #30182
Takaki et al. 2013
dTomato
psmyc
pHGFP
pUV15-pHGFP
6232 bp
Addgene #70045
Vandal et al. 2008
pJL29
6211bp
Addgene #227426
pJL30
6205 pb
Addgene #227427
B
pTEC15
pMV306-Hyg
pJL29
C
pTEC27
pJL30
D
Empty
7H9 Medium
pMV306-Hyg
pTEC15
pJL29
Bright light
Fluorescence
Merge
E
pTEC27
pJL30

Figure S2

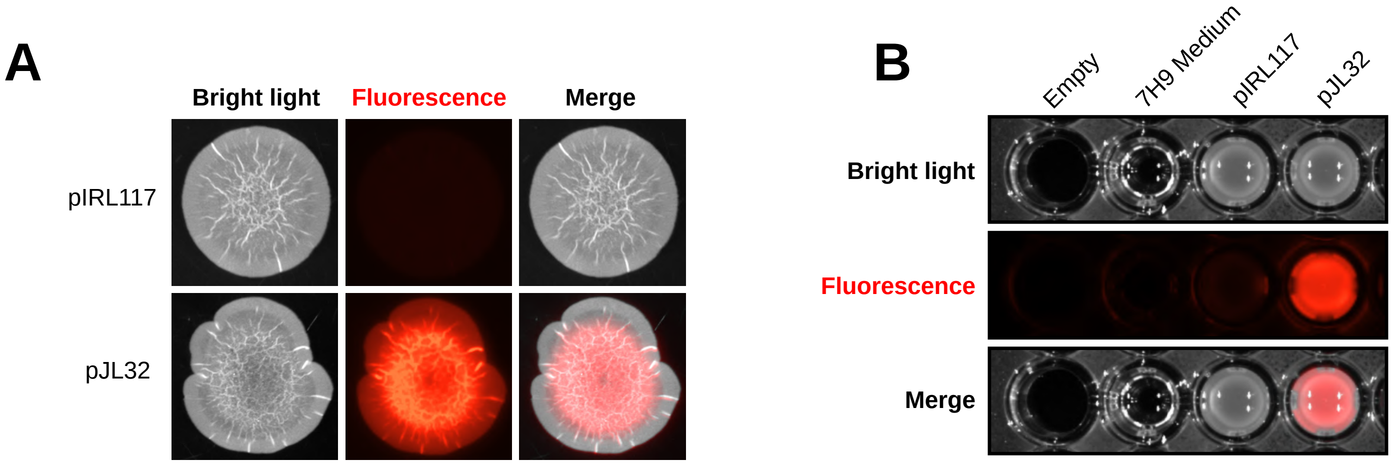
A
Bright light
Fluorescence
Merge
pIRL117
pJL32
B
Empty
7H9 Medium
pIRL117
pJL32
Bright light
Fluorescence
Merge

Figure S3

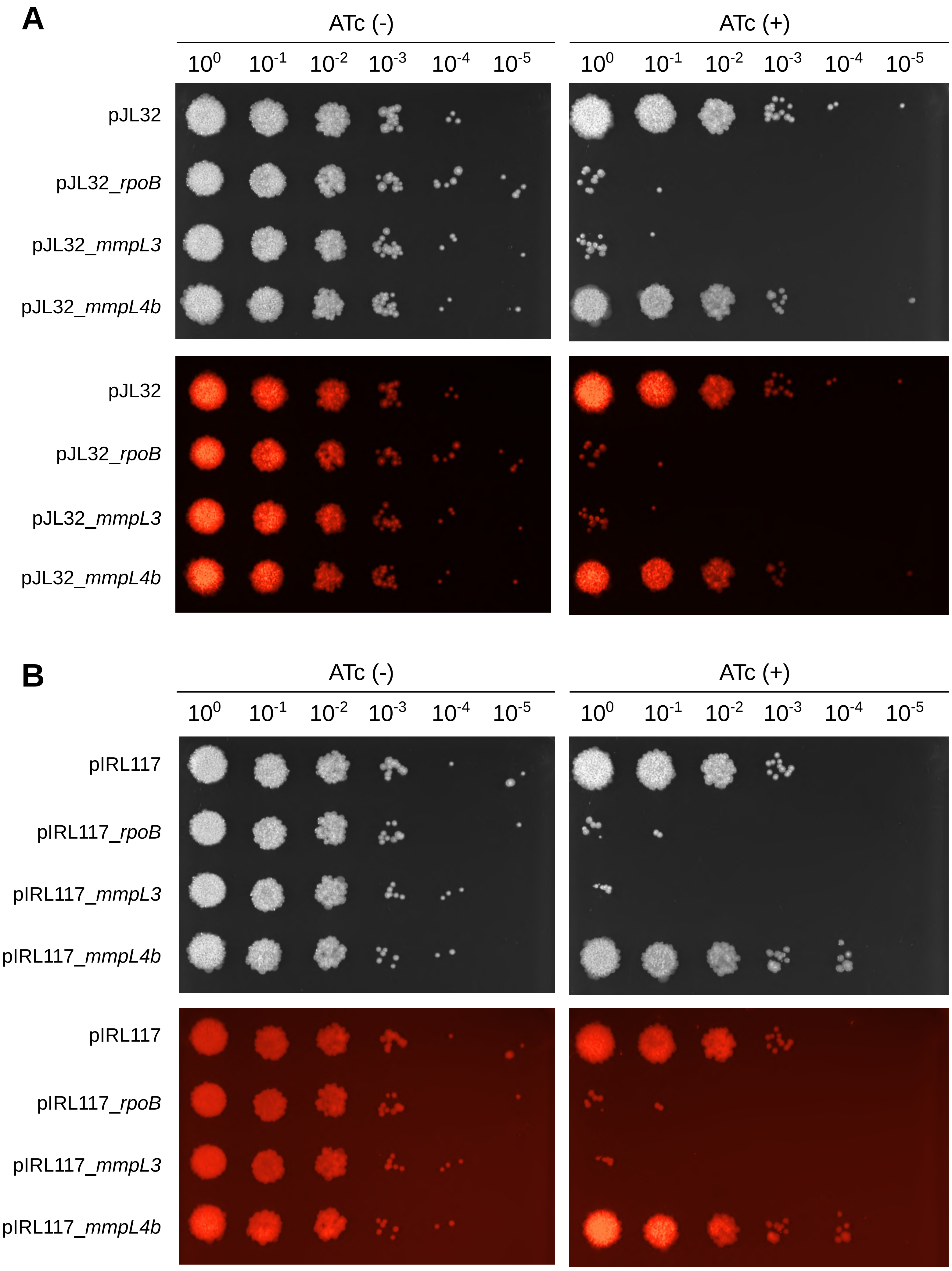
A
ATc (-)
ATc (+)
10^0 10^-1 10^-2 10^-3 10^-4 10^-5
pJL32
pJL32_rpoB
pJL32_mmpL3
pJL32_mmpL4b
B
pIRL117
pIRL117_rpoB
pIRL117_mmpL3
pIRL117_mmpL4b

Figure S4

A

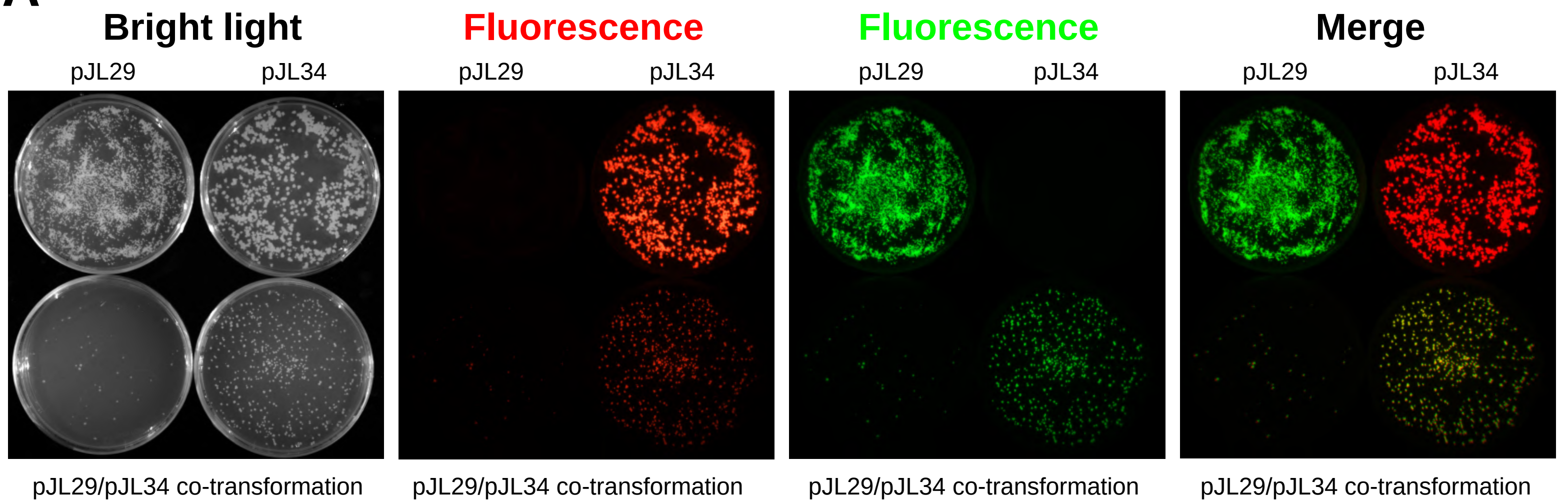
Bright light
Fluorescence
Fluorescence
Merge
pJL29
pJL34
pJL29
pJL34
pJL29
pJL34
pJL29
pJL34
pJL29/pJL34 co-transformation
pJL29/pJL34 co-transformation
pJL29/pJL34 co-transformation
pJL29/pJL34 co-transformation
